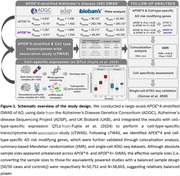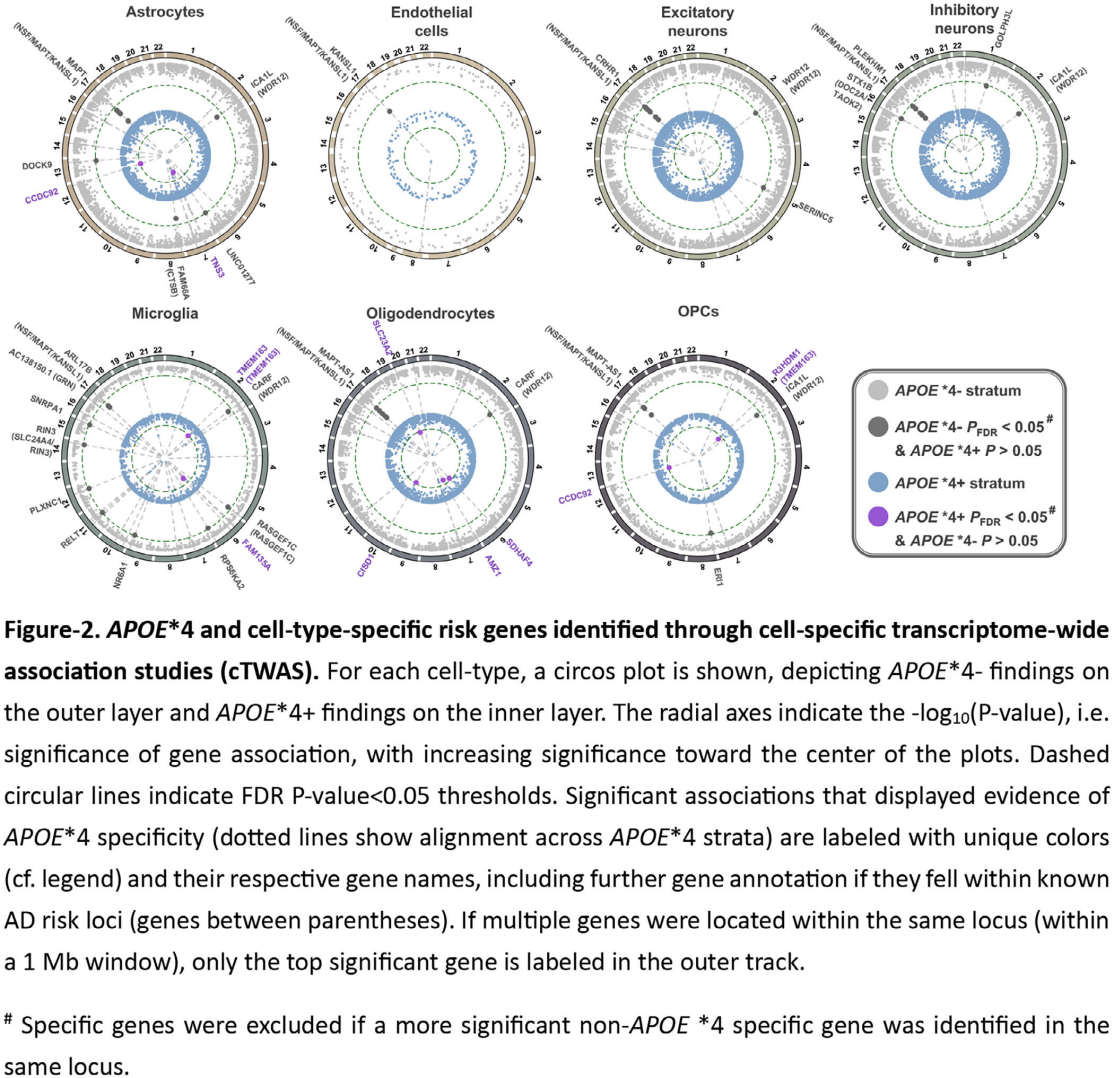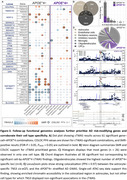# APOE*4 and Cell‐Type‐specific Risk‐Modifying Genes in Alzheimer’s Disease

**DOI:** 10.1002/alz70861_108597

**Published:** 2025-12-23

**Authors:** Youjie Zeng, Danielle M. Reid, Noah Cook, Chenyu Yang, Masashi Fujita, M. Ryan Corces, Vilas Menon, Michael D. Greicius, Michael E. Belloy

**Affiliations:** ^1^ NeuroGenomics and Informatics Center, Washington University School of Medicine, St Louis, MO USA; ^2^ Department of Neurology, Washington University School of Medicine, St Louis, MO USA; ^3^ Center for Translational and Computational Neuroimmunology, Department of Neurology, Columbia University Irving Medical Center, New York, NY USA; ^4^ Gladstone Institute of Neurological Disease, Gladstone Institutes, San Francisco, CA USA; ^5^ Department of Neurology, University of California San Francisco, San Francisco, CA USA; ^6^ Gladstone Institute of Data Science and Biotechnology, Gladstone Institutes, San Francisco, CA USA; ^7^ Department of Neurology and Neurological Sciences, Stanford University School of Medicine, Stanford, CA USA

## Abstract

**Background:**

APOE*4 has widespread effects on Alzheimer’s disease (AD) pathogenesis that can manifest through unique mechanisms across different cell types. Identification of cell‐type‐specific AD risk‐modifying genes according to an individual’s APOE*4 status may thus provide important therapeutic avenues. We sought to address this question through an approach integrating AD genetic association analyses with single‐cell multi‐omics data.

**Method:**

As outlined in Figure‐1, we performed large‐scale European ancestry APOE*4‐stratified genome‐wide association studies (GWAS) that we integrated with single‐nucleus expression quantitative trait loci (eQTLs) across seven brain cell types—astrocytes, endothelial cells, excitatory neurons, inhibitory neurons, microglia, oligodendrocytes, and oligodendrocyte progenitor cells (OPCs)—through cell‐type‐specific transcriptome‐wide association studies (cTWAS) using FUSION. Prioritized genes were further validated with Summary‐data‐based Mendelian Randomization (SMR), genetic colocalization analyses (COLOC), and single‐cell ATAC‐seq data.

**Result:**

We identified 61 gene–cell–APOE*4 combinations through cTWAS, including 10 APOE*4+ and 51 APOE*4− associations (Figure‐2). SMR and COLOC analyses validated 46 and 41 combinations, respectively, with 36 validated through both methods (Figure‐3A‐B). Most genes (*n* =26) showed APOE*4 specific associations in one cell type only (Figure‐3C), while the *MAPT* locus notably showed multiple APOE*4− gene associations across all 7 cell types (Figure‐3A). Endothelial cells, excitatory neurons, and inhibitory neurons yielded only APOE*4− loci, whereas astrocytes, microglia, oligodendrocytes, and OPCs revealed both APOE*4+ and APOE*4− loci (Figure‐3D). Notably, oligodendrocytes displayed the largest fraction of APOE*4+ loci across cell‐types. An interesting observation was that *TNS3* expression in astrocytes was inversely associated with AD risk in APOE*4+ individuals, supported by strong colocalization (PP4=0.97), SMR, and increased chromatin accessibility around the *TNS3* eQTL signal in astrocytes compared to other cell types (Figure‐3E). A recent study reported that *TNS3* mediates connections between glioblastoma multiforme and AD transcriptomic networks, while further observing that *TNS3* and *APOE* levels were strongly, inversely correlated. This provides potential mechanistic insight into the link of *TNS3* to AD risk, which will be a further point of investigation.

**Conclusion:**

We identified multiple AD risk‐modifying genes dependent on both cellular context and APOE*4 status. These insights may inform therapeutic strategies aimed at APOE*4 and guide experimental interrogation of *APOE*’s pathogenic mechanisms across brain cell types.